# Longitudinal association between movement behaviours and depressive symptoms among adolescents using compositional data analysis

**DOI:** 10.1371/journal.pone.0256867

**Published:** 2021-09-01

**Authors:** Hugues Sampasa-Kanyinga, Ian Colman, Dorothea Dumuid, Ian Janssen, Gary S. Goldfield, Jian Li Wang, Karen A. Patte, Scott T. Leatherdale, Jean-Philippe Chaput

**Affiliations:** 1 School of Epidemiology and Public Health, University of Ottawa, Ottawa, Ontario, Canada; 2 Healthy Active Living and Obesity Research Group, Children’s Hospital of Eastern Ontario Research Institute, Ottawa, Ontario, Canada; 3 Centre for Fertility and Health, Norwegian Institute of Public Health, Oslo, Norway; 4 Allied Health & Human Performance, Alliance for Research in Exercise, Nutrition and Activity (ARENA), University of South Australia, Adelaide, Australia; 5 School of Kinesiology and Health Studies, Queen’s University, Kingston, Ontario, Canada; 6 University of Ottawa Institute of Mental Health Research, Ottawa, Ontario, Canada; 7 Department of Health Sciences, Faculty of Applied Health Sciences, Brock University, Niagara Region, St. Catharines, Ontario, Canada; 8 School of Public Health and Health Systems, University of Waterloo, Waterloo, Ontario, Canada; University of Maiduguri College of Medical Sciences, NIGERIA

## Abstract

**Background:**

Research examining the associations between movement behaviours and mental health indicators within a compositional framework are sparse and limited by their cross-sectional study design. This study has three objectives. First, to describe the change in movement behaviour composition over time. Second, to explore the association between change in movement behaviour composition and change in depressive symptoms. Third, to explore how reallocations of time between movement behaviours are associated with changes in depressive symptoms.

**Methods:**

Longitudinal data of 14,620 students in grades 9–12 (mean age: 14.9 years) attending secondary schools in Canada (Ontario, British Columbia, Alberta, Quebec) were obtained from two waves (2017/18, 2018/19) of the COMPASS study. Moderate-to-vigorous physical activity (MVPA), recreational screen time, and sleep duration were self-reported. Depressive symptoms were measured using the Center for Epidemiologic Studies Depression Scale (Revised)−10 (CESD-R-10). Compositional data analyses using pivot coordinates and compositional isotemporal substitution for longitudinal data were used to analyse the data. Analyses accounted for school clustering, were stratified by gender and age (< or ≥ 15 years), and were adjusted for race/ethnicity, body mass index z-score, baseline movement behaviour composition, and baseline depressive symptoms.

**Results:**

There were significant differences in movement behaviour composition over time across all subgroups. For example, the relative contributions of MVPA and sleep duration to the movement behaviour composition decreased over time while screen time increased among younger boys and girls and older girls. Increasing sleep duration relative to the remaining behaviours (i.e. screen time and MVPA) was associated with lower depressive symptoms among all subgroups. Increasing screen time relative to the remaining behaviours (i.e. MVPA and sleep duration) was associated with higher depressive symptoms among all subgroups. Increasing MVPA relative to the remaining behaviours (i.e. screen time and sleep duration) was associated with lower depressive symptoms in older girls only. Isotemporal substitution estimates indicated that decreasing screen time by 60 minutes/day and replacing that time with 60 minutes of additional sleep is associated with the largest change in depressive symptoms across all subgroups.

**Conclusions:**

Findings from this prospective analysis suggest that increased sleep duration and reduced screen time are important determinants of lower depressive symptoms among adolescents.

## Introduction

Adolescence is a sensitive period in the lifespan. It is the period of transition between childhood and adulthood, marked by important biological changes during puberty and the heightened influence of the social context in which young people are growing up [1,2]. As adolescents become more independent, they become more responsible for making their own lifestyle choices [2,3]. It is during this developmental period that more divergence in lifestyle behaviours and mental health outcomes emerge [4]. It is therefore important to examine how developmental changes in health behaviours impact health outcomes, in order to intervene before those behaviours become ingrained.

Research has shown that half of mental health problems have their onset during adolescence, particularly by age 14 [4]. It is estimated that 10 to 20% of adolescents globally experience mental health problems [5]. Depression is one of the most common forms of mental disorders that adolescents experience [5]. It is a leading cause of disability worldwide and a major contributor to the overall global burden of disease [5]. Adolescent depression is associated with long-term negative outcomes in adulthood [6,7]. A recent study has indicated that 8% of Ontario (Canada) youth aged 12 to 17 years had symptoms that met criteria for major depression in 2014 [8]. A US study in adolescents reported that the prevalence of depression changed from 8.7% in 2005 to 11.3% in 2014 [9], indicating that the prevalence of depression has increased over the past years. Experience of depression increases with age, and more girls are affected by depression than boys [10–12]. Research is needed to identify modifiable risk factors of depression among adolescents as the results from such research would provide insights into the design of interventions that could reduce depression incidence.

Movement behaviours, including physical activity, sedentary behaviours, and sleep are associated with changes in depressive symptoms across adolescence [13,14]; however, research that looks at these behaviours concurrently is sparse, even though it is evident that these movement behaviours are intrinsically linked. It is not possible to increase time spent in one behaviour without equivalent reduction in time spent across the remaining behaviours, because the day only has 24 hours. Compositional data analysis is an appropriate analytical approach to deal with data that is a proportion of a finite total, and can be used when all components or just some components of the finite total have been measured [15]. Compositional data analyses use the correct geometry (i.e., closed space versus open space) for bounded data and findings are interpreted as the effects of a behaviour as a proportion relative to the other behaviours instead of a behaviour being independent of another behaviour [15,16]. To date, compositional data analyses have been mostly used to examine the health implications of sleep duration, sedentary time, and physical activity on obesity and cardio-metabolic health markers in both adults and children [15–17]. Very few studies have used such methodology in relation to mental health outcomes in children and adolescents specifically [18]. Furthermore, previous studies are limited either by their cross-sectional designs and/or small sample sizes [18]. Studies examining the prospective relationships between movement behaviours (sleep duration, sedentary time, and physical activity) and mental health indicators in adolescents using compositional analyses are thus warranted.

The isotemporal substitution model was developed as a modelling strategy that can be used to estimate the effects of substituting time from one movement behaviour with an equal amount of time from another movement behaviour [19]. For instance, a recent study investigating reallocating time between sleep, sedentary behaviour, and physical activity among adults generally found beneficial effects of replacing time spent sedentary or sleeping with physical activity for reducing mortality risk [20]. Studies that have examined the isotemporal substitution between physical activity, sedentary time and sleep duration on specific mental health outcomes such as depressive symptoms are sparse, particularly among children and adolescents. Indeed, a previous systematic review of studies that employed isotemporal substitution model in sleep, sedentary behaviour, and physical activity research has indicated that only three studies have been conducted in relation to mental health outcomes; two among adults and one in adolescents [21]. In a study of more than 20,000 children from the Canadian Health Behavior in School-aged Children study, Janssen [22] used isotemporal substitution models to estimate whether replacing time spent in sedentary video games and active outdoor play with active video games was associated with changes in youth’s mental health. His results showed that replacing sedentary video games with active video games was associated with better mental health, whereas replacing active outdoor play with active video games was associated with more deleterious mental health indicators [22]. However, this study did not examine other types of movement behaviours such as sleep duration, total recreational screen time or physical activity, and also did not account for the compositional properties of time-use data [23,24]. Furthermore, this study was limited by a cross-sectional design, thus supporting the need for prospective studies to confirm temporality.

Within a compositional framework, the present study has three objectives. First, to describe change in adolescents’ movement behaviour composition over time. Second, to explore the association between change in movement behaviour composition and change in depressive symptoms during adolescence. Third, to explore how reallocations of time between movement behaviours are associated with changes in depressive symptoms during adolescence.

## Methods

### Design

The COMPASS study is a prospective cohort study (2012–2021) collecting longitudinal hierarchical data at the student, school and community levels in Ontario, British Columbia, Alberta and Quebec (Canada) to understand how changes in school characteristics (policies, programs, built environment) are associated with changes in youth health behaviours [25]. A large convenience sample of schools that allow active-information passive-consent protocols were recruited and eligible students in those schools participate in the COMPASS survey during class time once annually. All students present during data collection were eligible to complete the questionnaire during class, enabling collection of whole-school samples. All student-level data were collected through a paper-based questionnaire comprising questions on many health, social, and academic outcomes. The questionnaire underwent and performed well in validity and reliability testing [26,27]. This paper uses student-level data from Year 6 (2017/18) and Year 7 (2018/19) of the COMPASS host study (www.compass.uwaterloo.ca) [25]. COMPASS received ethics approval from the University of Waterloo Human Research Ethics Committee (ORE #: 30118) and all participating school boards. All students attending participating schools were invited to participate using active-information passive-consent parental permission protocols. Students could withdraw from the study at any time.

Data from schools that participated in both Year 6 (2017/18) and Year 7 (2018/19) of COMPASS were used. In Year 6, 66,501 grade 9–12 students from 124 schools (8 in Alberta, 16 in British Columbia, 61 in Ontario, 37 in Quebec, and 2 in Nunavut) participated and in Year 7, 74,501 students from 136 secondary schools (8 in Alberta, 15 in British Columbia, 61 in Ontario, and 52 in Quebec) participated. Student participation rates for Year 6 and 7 of the COMPASS study was 81.9% and 84.2%, respectively. Each school was assigned a unique identifier, which was used to link the school samples across waves. Unique self-generated identification codes are used to link student-level data sets across different waves [28]. Reasons for non-linkage included students graduating or being newly admitted to school within the waves, students transferring schools, having a spare/free period or being otherwise absent during data collection, dropping out of school, or inaccurate data provided on the data linkage measures. Detailed information on the COMPASS design and methodology are available elsewhere [25].

### Dependent variable

Depressive symptoms were assessed using the Center for Epidemiologic Studies Depression Scale (Revised)−10 (CESD-R-10) [29]. This 10-item scale was designed to assess self-reported symptoms of depression such as feelings of sadness, hopelessness, apathy and motivation, irritability, and difficulties sleeping, making decisions, and concentrating over a 1-week period. The CESD-R-10 has shown strong psychometric properties in a sample of adolescents, with good model fit of a 1-factor model [30]. All questions include four response categories (0–3). The total score was calculated by summing all 10 items, with higher scores indicating higher levels of depressive symptoms. Internal consistency of the CESD-R-10 scale was high (α = 0.98).

### Independent variables

Independent variables included moderate-to-vigorous intensity physical activity (MVPA), recreational screen time, and sleep duration. MVPA was measured using items that asked students how many minutes of moderate physical activity (defined in the survey as lower intensity activities, in comparison to vigorous physical activity, such as walking, biking to school, and recreational swimming) and how many minutes of hard physical activity (i.e. vigorous–defined as physical activities that increase their heart rate and make them breathe hard and sweat) they had done in the past seven days. Students reported the number of hours and minutes spent in each intensity level of physical activity for each day in the previous week. Average daily duration of MVPA in minutes was calculated as: (5 * MVPA on weekdays) + (2 * MVPA on weekend days)/7. Recreational screen time was measured using an item that asked students how much time per day they usually spend doing the following activities: watching/streaming TV shows or movies, playing video/computer games, surfing the internet, and texting/messaging/emailing. Students reported their screen time in hours and minutes. Time duration from each of the 4 activities were summed to represent total average daily recreational screen time. Sleep duration was measured using an item that asked students how much time per day they usually spend sleeping. Students reported their sleep duration in hours and minutes.

#### Covariates

Age (years), gender (boy/girl), race/ethnicity (White/Black/Asian/Latin American/Hispanic/Other), body mass index (BMI) z-score, baseline movement behaviour, and baseline depressive symptoms were used as covariates. BMI was calculated using self-reported height and weight. BMI z-scores were computed according to the World Health Organization’s reference data [31].

### Data processing

**Fig 1:**
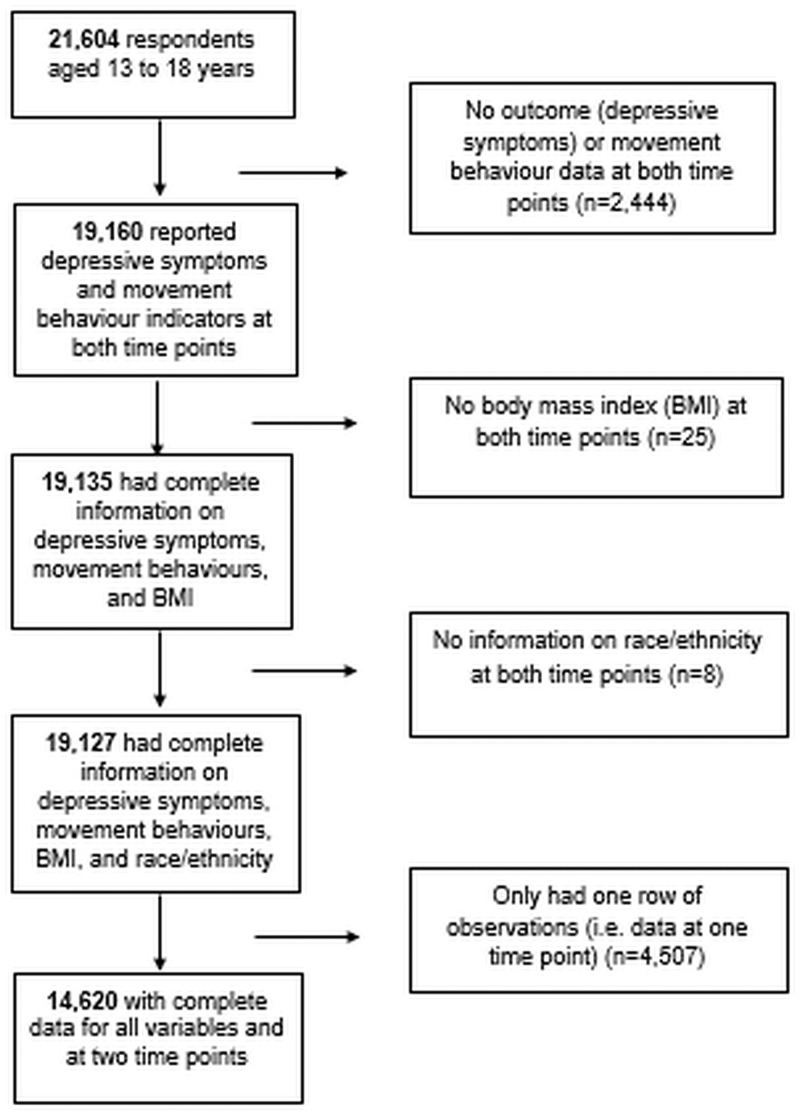
Participant flow-diagram.

Of the 21,604 students who were successfully linked for their participation in COMPASS in Year 6 (2016/17, considered herein as the baseline data) and Year 7 (2018/19, considered herein as the follow-up data), 19,160 answered questions regarding depressive symptoms and movement behaviours at both time points (Fig 1). Of those, 14,620 students had complete data for all variables included in our analyses at both time points and constituted our analytical sample.

Gender at time 2 was replaced by gender at time 1 if participants changed gender (n = 21). A total of 1,156 respondents reclassified race/ethnicity or had not reported race/ethnicity for one time point. Of those, 30 did not report race/ethnicity at time 1. Eight out of those 30 were excluded because they had no information on race/ethnicity at both time points. Race/ethnicity at time 1 was replaced by race/ethnicity at time 2 if participants did not report race/ethnicity at time 1 (n = 22). Whereas, race/ethnicity at time 2 was replaced by race/ethnicity at time 1 if participants changed race/ethnicity (n = 1,126). Time use across the three movement behaviours was adding up to greater than 24-hours per day. Therefore, movement behaviour variables were checked for outliers (≥ ± 2 standard deviations) and truncation was performed for all daily duration values exceeding +2 standard deviations (SD) for MVPA and screen time and -2 SD for sleep duration to avoid implausible data.

### Statistical analyses

All analyses were carried out in R using the Compositions [32], zCompositions [33], lme4 [34] and ggtern [35] packages.

#### Change in movement behaviour composition (1^st^ objective)

Repeated-measures multivariate analysis of variance (MANOVA) models with isometric log-ratios (ilrs) as dependent variables and time-point as the independent variable were used to determine whether the movement behaviour composition changed between the two time-points [36]. Interactions between time-point and age and time-point and gender were tested. Given that these were significant (p<0.001), all subsequent analyses were stratified by gender and age (median split at 15 years). The repeated-measures MANOVA indicates whether within-participant composition changed between the time points but provides no information about which behaviours were driving the changes. To explore which behaviours were driving the changes, we calculated the mean within-person log-ratio change for each behaviour; for example, *ln*(Sleep^Time2^ / Sleep^Time1^) for each participant, and then averaged across the strata. Bootstrapping with 1000 replicates generated 95% confidence intervals for each mean log-ratio difference.

#### Association between change in movement behaviour composition and change in depressive symptoms (2^nd^ objective)

The movement behaviour composition was expressed as a specific type of *ilr* previously called pivot coordinates [37]. Briefly, pivot coordinates are a set of *ilrs* where the first coordinate enables one part of the composition (e.g., sleep) to be considered relative to the remaining parts of the composition (i.e., sedentary time and MVPA) [15,38]. Sleep is in the numerator of the log-ratio while the remaining parts are in the denominator. We created three sets of pivot coordinates to enable each behaviour (sleep, sedentary behaviour, and MVPA) to be considered relative to the remaining behaviours. We used the change in pivot coordinates as the explanatory variable in mixed effects multiple linear regression models. The dependent variable was change in depressive symptoms. The models were run, one for each set of pivot coordinates. The models also included covariates of time 1 composition (expressed as *ilrs*), time 1 depressive symptoms, time 1 BMI z-score and race/ethnicity. A random intercept for school was used to account for the school-based sampling frame. A Wilks’ ANOVA test of the mixed effects multiple linear regression models was used to determine whether the change in movement behaviour composition was associated with change in depressive symptoms. The standardized beta coefficients for change in each pivot coordinate (one for each behaviour, relative to remaining behaviours) from each of the three models was presented in a table to describe how change in one behaviour (relative to the remaining behaviours) was associated with change in depressive symptoms.

We used the mixed effects multiple linear regression model to predict change in depressive symptoms for 10,000 random hypothetical time 2 compositions within the empirical ranges observed in the sample. The hypothetical time 2 compositions were considered relative to the centre of the time 1 composition. As in Olds et al. [39], the estimated change in depressive symptoms associated with no change in time-use composition was subtracted from the predictions to isolate change in depressive symptoms associated with change in time-use only. Estimated changes in depressive symptoms were expressed as effect sizes, using pooled standard deviations of time 1 and time 2 depression scores. Estimated change in depressive symptoms were colour-coded within ternary diagrams so that yellow indicates no change from time 1 depressive symptoms, increases in depressive symptoms are coloured orange-to-red, and decreases are coloured green-to-blue.

#### Compositional isotemporal substitution (3^rd^ objective)

Using compositional isotemporal substitution [40] for longitudinal data [41], we estimated the change in depressive symptoms (presented as standardized effect sizes) associated with reallocating 60 minutes between movement behaviours around the centre time 1 composition. The centre time 1 movement behaviour composition was linearly adjusted so that the behaviours summed to the mean total time of 957 minutes. Hypothetical situations of all possible reallocations of 60 minutes to/from the time 1 centre composition were used as new data for prediction in the mixed effects multiple linear regression models. Again, estimated change in depressive symptoms for no change in composition was subtracted to isolate change in depressive symptoms associated with changes in composition only. We derived 95% confidence intervals (CI) for the estimated difference in depressive symptoms using the model-estimated standard error of the difference.

## Results

Characteristics of the study sample at time 1 and time 2 are presented in Table 1. At time 1, the mean age was 14.9 years and most participants identified themselves as White (73.3%). The average body weight status was within normal range in the total sample and across all age/gender groups. In the total sample and across all subgroups, sleep duration represented the greatest portion of the composition, followed by screen time, and far behind was MVPA.

**Table 1:** Descriptive characteristics of the study sample.

Characteristics	Total population	Younger boys	Younger girls	Older boys	Older girls
**2017 sample**	N = 14,620	N = 2,836	N = 2,264	N = 5,060	N = 4,460
Age (mean (SD))	14.92 (1.15)	13.62 (0.64)	13.65 (0.62)	15.59 (0.66)	15.62 (0.69)
Race/ethnicity (%)					
White	10,722 (73.3)	2,168 (76.4)	1,774 (78.4)	3,621 (71.6)	3,159 (70.8)
Black	392 (2.7)	67 (2.4)	70 (3.1)	119 (2.4)	136 (3.0)
Asian	1,723 (11.8)	262 (9.2)	152 (6.7)	726 (14.3)	583 (13.1)
Latin American/Hispanic	395 (2.7)	69 (2.4)	55 (2.4)	135 (2.7)	136 (3.0)
Other	1,388 (9.5)	270 (9.6)	213 (9.4)	459 (9.1)	446 (10.0)
BMI (mean (SD))	21.43 (4.05)	20.45 (3.90)	20.67 (4.00)	21.66 (3.87)	22.17 (4.17)
BMI z-score (mean (SD))	0.25 (0.99)	0.26 (1.01)	0.22 (0.96)	0.21 (1.02)	0.31 (0.94)
Depressive symptoms score (mean (SD))	8.05 (5.76)	8.22 (5.87)	5.89 (4.48)	9.81 (6.26)	7.04 (5.02)
Depressive symptoms z-scores (mean (SD))	-0.04 (0.99)	-0.01 (1.01)	-0.41 (0.77)	0.26 (1.07)	-0.22 (0.86)
**Average overall sum of times recorded for each person (%)**					
MVPA	9.5	9.2	9.2	8.6	8.6
Screen time	40.1	36.8	36.8	41.8	41.8
Sleep duration	50.4	53.9	53.9	49.7	49.7
**2018 sample**	N = 14,620	N = 2,836	N = 2,264	N = 5,060	N = 4,460
Age (mean (SD))	15.91 (1.15)	14.62 (0.68)	14.67 (0.66)	16.58 (0.67)	16.61 (0.70)
Race/ethnicity (%)					
White	10722 (73.3)	2168 (76.4)	1774 (78.4)	3621 (71.6)	3159 (70.8)
Black	392 (2.7)	67 (2.4)	70 (3.1)	119 (2.4)	136 (3.0)
Asian	1723 (11.8)	262 (9.2)	152 (6.7)	726 (14.3)	583 (13.1)
Indigenous	553 (3.8)	87 (3.1)	78 (3.4)	200 (4.0)	188 (4.2)
Latin American/Hispanic	395 (2.7)	69 (2.4)	55 (2.4)	135 (2.7)	136 (3.0)
Other	835 (5.7)	183 (6.5)	135 (6.0)	259 (5.1)	258 (5.8)
BMI (mean (SD))	21.99 (4.03)	21.08 (3.62)	21.47 (4.08)	22.05 (3.87)	22.76 (4.26)
BMI z-scores (mean (SD))	0.23 (0.99)	0.27 (0.96)	0.27 (0.95)	0.11 (1.06)	0.32 (0.95)
Depression (mean (SD))	8.83 (5.99)	9.72 (6.32)	6.78 (5.14)	10.30 (6.22)	7.63 (5.33)
Depression z-scores (mean (SD))	-0.00 (1.00)	0.15 (1.06)	-0.34 (0.86)	0.25 (1.04)	-0.20 (0.89)
**Average sum of times recorded for each student (%)**					
MVPA	8.3	8.2	8.2	7.2	7.2
Screen time	42.0	39.4	39.4	42.7	42.7
Sleep duration	49.7	52.4	52.4	50.2	50.2

BMI, body mass index; MVPA, moderate-to-vigorous physical activity; SD, standard deviation.

### Change in movement behaviour composition (1^st^ objective)

**Fig 2:**
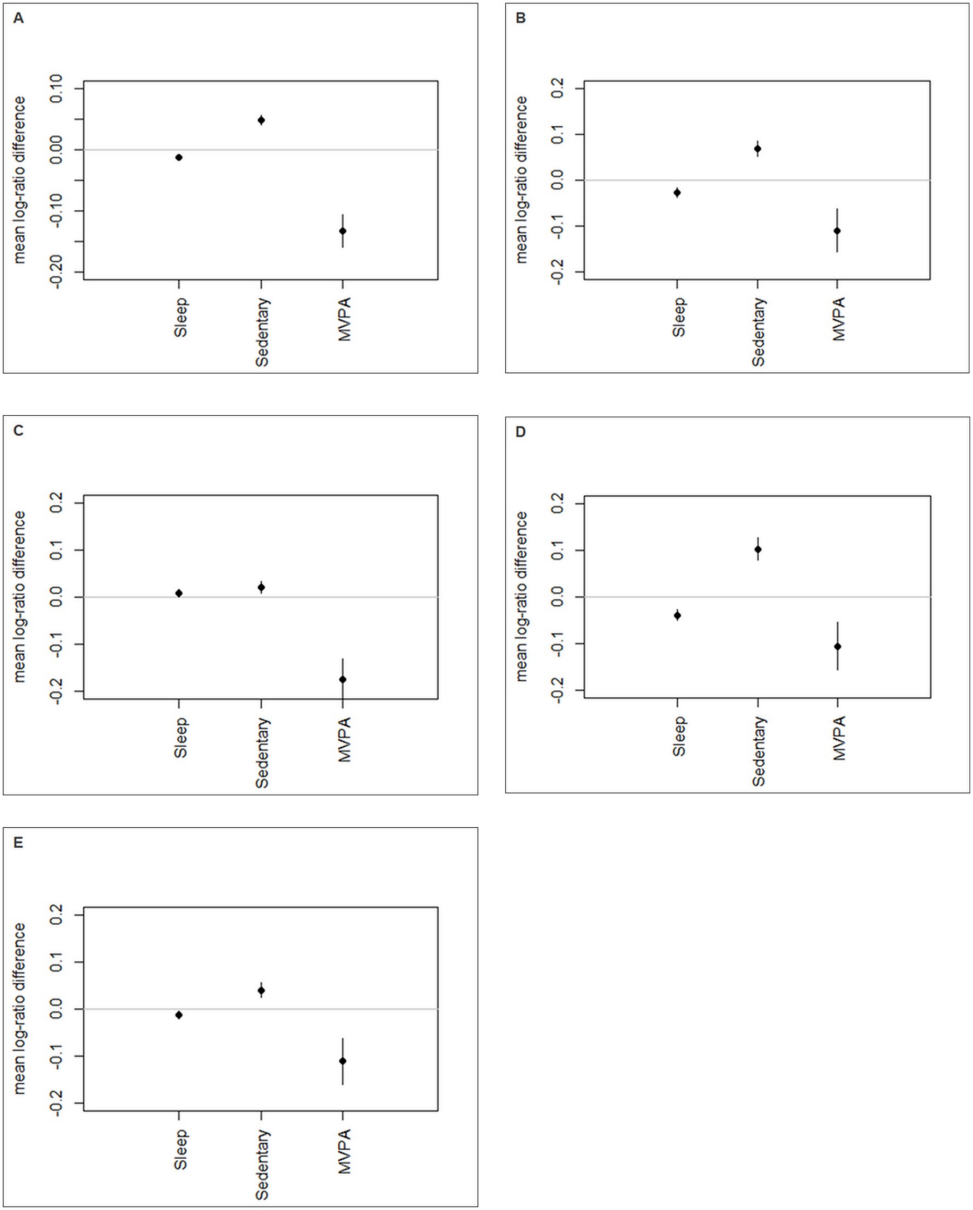
Change in movement behaviours over time. **Note:** Graphs display the mean log-ratio difference plots and their 95% confidence intervals for change over time of movement behaviours in the total sample (Panel A, n = 14,620), younger boys (Panel B, n = 2,836), older boys (Panel C, n = 2,264), younger girls (Panel D, n = 5,060), and older girls (Panel E, n = 4,460). MVPA, moderate-to-vigorous physical activity. These changes reflect changes in the relative contribution to the movement behaviours to the overall movement behaviour contribution.

Results from analyses examining whether the movement behaviour composition changed between the two time points are outlined in Table 2. Overall, there were significant intra-individual relative differences in movement behaviour composition over time in the total sample and in all age/gender groups (p<0.001). Results further indicated that all the contribution of each component to the overall movement behaviour composition varied significantly from time 1 to time 2. For example, in the overall sample, the relative contribution of MVPA to the movement behaviour composition decreased by 13% (estimate: -0.133; 95% CI: -0.107;-0.159), the relative contribution of screen time increased by 5% (estimate: 0.048; 95% CI: 0.056;0.041), and the relative contribution of sleep duration decreased by 1% (estimate: -0.012; 95% CI: -0.007;-0.017) over time (Fig 2A). Patterns were similar among younger boys (Fig 2B), younger girls (Fig 2D), and older girls (Fig 2E). However, among older boys, MVPA decreased by 17% (estimate: -0.174; 95% CI: -0.130; -0.221) (Fig 2C), whereas sleep duration increased by 1% (estimate: 0.008;95% CI: 0.001; 0.016) and screen time increased by 2% (estimate: 0.021; 95% CI: 0.007; 0.034) over time.

**Table 2:** Differences in movement behaviour composition over time.

Characteristics	Total population (N = 14,620)	Younger boys (N = 2,836)	Older boys (N = 2,264)	Younger girls (N = 5,060)	Older girls (N = 4,460)
**Overall difference in composition over time**					
*F*	106.200	37.688	30.376	42.628	22.447
*df*	2, 146	2, 283	2, 506	2, 226	2, 446
*p*-value	<0.001	<0.001	<0.001	<0.001	<0.001
**Mean log-ratio difference in behaviours over time** *					
MVPA	-0.133 (-0.107; -0.062)	-0.112 (-0.062; -0.157)	-0.174 (-0.130; -0.221)	-0.107 (-0.054; -0.156)	-0.111 (-0.160; -0.06)
Screen time	0.048 (0.041; 0.056)	0.069 (0.052; 0.085)	0.021 (0.007; 0.034)	0.102 (0.079; 0.126)	0.040 (0.026; 0.057)
Sleep duration	-0.012 (-0.007; -0.017)	-0.028 (-0.018; -0.038)	0.008 (0.001; 0.016)	-0.039 (-0.051; -0.027)	-0.012 (-0.020; -0.004)

df: Degrees of freedom; MVPA: Moderate-to-vigorous physical activity.

*These changes reflect changes in the relative contribution of the movement behaviours to the overall movement behaviour composition. A log-ratio difference of -0.133 indicates an average within-person reduction of 13.3% between the two time-points. That means it is taking up 13% less of the total time spent in MVPA + screen time + sleep duration.

### Association between change in movement behaviour composition and change in depressive symptoms (2^nd^ objective)

Table 3 summarizes results of analyses examining the longitudinal associations between changes in pivot log-ratio coefficients and follow-up depressive symptoms. Before and after adjusting for covariates, the beta estimates for the pivot coordinates indicated that increasing sleep duration relative to remaining behaviours (i.e. screen time and MVPA) was associated with lower depressive symptoms among all subgroups. Conversely, increasing screen time relative to the remaining behaviours (i.e. MVPA and sleep duration) was associated with higher depressive symptoms among all age/gender groups. There was no association between MVPA, relative to the remaining behaviours, and depressive symptoms among younger and older boys and younger girls. However, among older girls, increasing MVPA relative to the remaining behaviours (i.e. screen time and sleep duration) was associated with lower depressive symptoms.

**Table 3:** Longitudinal associations between changes in first pivot coordinate coefficient (standardized betas) and change in depressive symptoms.

Models^a^	Younger boys (N = 2,836)		Older boys (N = 2,264)		Younger girls (N = 5,060)		Older girls(N = 4,460)	
	Std_β (SE)	p value	Std_β (SE)	p value	Std_β (SE)	p value	Std_β (SE)	p value
Model 1								
Sleep vs. remaining	-0.196 (0.025)	<0.001	-0.209 (0.022)	<0.001	-0.215 (0.028)	<0.001	-0.154 (0.023)	<0.001
Screen time vs. remaining	0.218 (0.026)	<0.001	0.233 (0.023)	<0.001	0.241 (0.031)	<0.001	0.224 (0.024)	<0.001
MVPA vs. remaining	0.011 (0.020)	0.577	0.002 (0.014)	0.906	0.034 (0.023)	0.143	-0.081 (0.015)	<0.001
Model 2								
Sleep vs. remaining	-0.196 (0.025)	<0.001	-0.208 (0.022)	<0.001	-0.216 (0.028)	<0.001	-0.151 (0.023)	<0.001
Screen time vs. remaining	0.218 (0.026)	<0.001	0.230 (0.023)	<0.001	0.243 (0.031)	<0.001	0.221 (0.024)	<0.001
MVPA vs. remaining	0.011 (0.020)	0.578	0.004 (0.014)	0.792	0.033 (0.023)	0.153	-0.080 (0.015)	<0.001

Std_β: Standardized beta; SE: Standard error.

^a^All models are adjusted for 2017 movement behaviour composition and baseline depressive symptoms. Model 2 is further adjusted for race/ethnicity and time 1 BMI z-scores.

**Fig 3:**
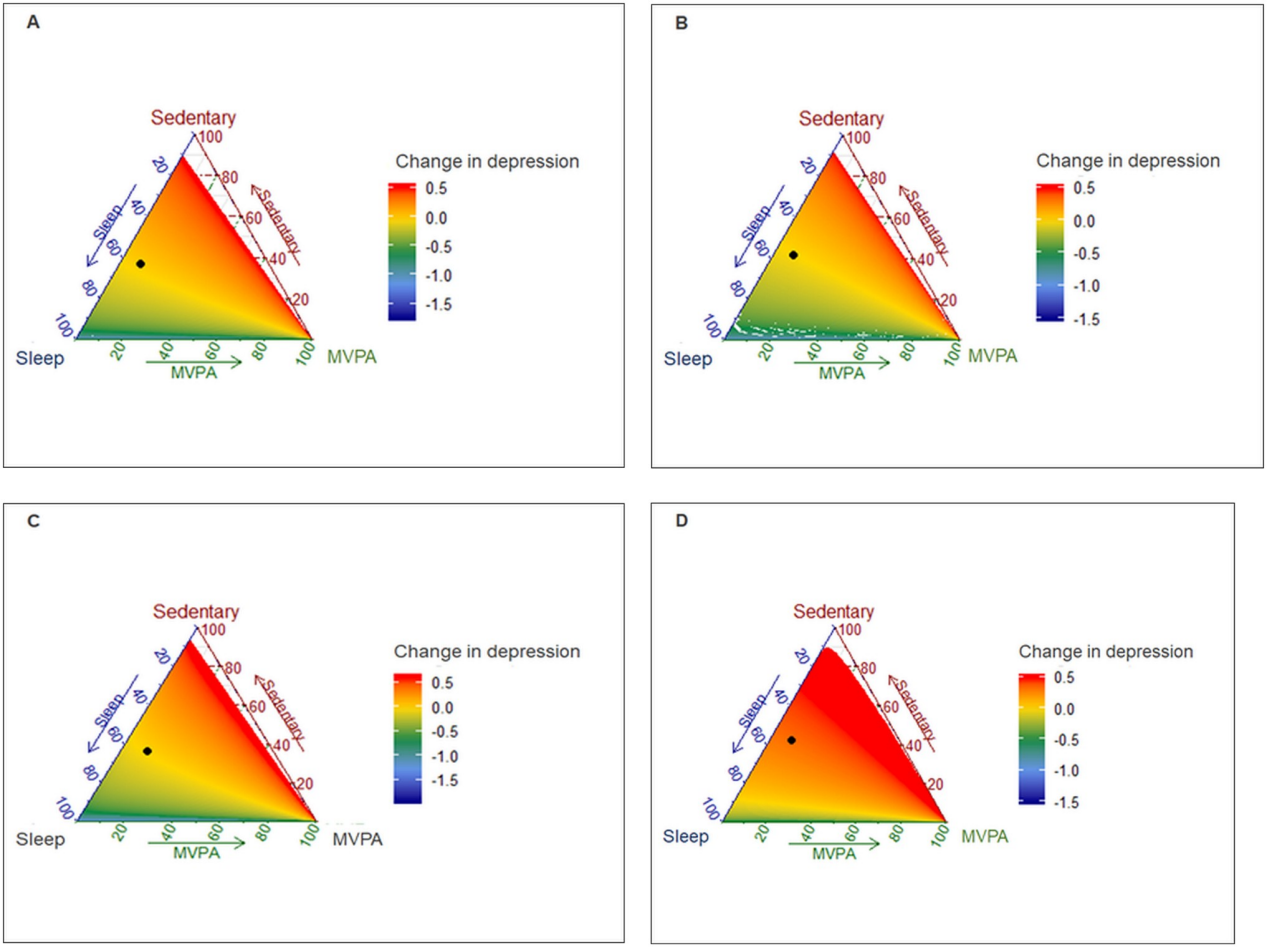
Data frame showing the predictive changes in composition and the predicted change in depressive symptoms. (A) Younger boys (n = 2,836); (B) Older boys (n = 2,264); (C) Younger girls (n = 5,060); (D) Older girls (n = 4,460). Black dot represents mean composition at time 1. The datapoints are color-coded according to their model-predicted change in depressive symptoms such that yellow = no change, red = increased depressive symptoms, blue = decreased depressive symptoms. Change away from the centre (mean composition at time 1) towards each activity informs the presence or absence of variation in color, which suggest no change, increased or decreased depressive symptoms.

Color-coding the datapoints according to their model-predicted change in depressive symptoms (yellow = no change, red = increased depressive symptoms, blue = decreased depressive symptoms) shows that a change away from the centre (mean composition at time 1) towards physical activity is flat, indicating no variation in color for the total sample and all subgroups, except older girls (Fig 3). This suggests that a change towards MVPA did not change depressive symptoms for younger boys, older boys, and younger girls. However, among older girls, increasing MVPA while equally reducing screen time and sleep duration was associated with decreased depressive symptoms. A change away from the mean composition at time 1 towards screen time was associated with increased depressive symptoms across all subgroups. Finally, increasing sleep duration while equally reducing MVPA and screen time was associated with decreased depressive symptoms.

### Compositional isotemporal substitution (3^rd^ objective)

Estimated changes in follow-up depressive symptoms associated with 60-minute reallocations from the mean baseline movement behaviour composition are presented in Table 4. Replacing 60 minutes of screen time by 60 minutes of either sleep duration or MVPA was associated with decreased depressive symptoms in all age/gender groups. In contrast, replacing 60 minutes of sleep duration by 60 minutes of either MVPA or screen time was associated with increased depressive symptoms in all age/gender groups. Similarly, replacing 60 minutes of MVPA by 60 minutes of screen time was associated with increased depressive symptoms in all age/gender groups. However, replacing 60 minutes of MVPA by 60 minutes of sleep duration was associated with lower depressive symptoms in all age/gender groups, except older girls, for whom such a time reallocation was associated with increased depressive symptoms. It is noteworthy that predicted changes in depressive symptoms were strongest and most beneficial when replacing 60 minutes of screen time by 60 minutes of sleep duration across all subgroups, as it had the lowest coefficient for depressive symptoms.

**Table 4:** Estimated changes in follow-up depressive symptoms associated with 60-minute reallocations from the mean baseline movement behaviour composition*.

	Younger boys (N = 2,836)	Older boys (N = 2,264)	Younger girls (N = 5,060)	Older girls (N = 4,460)
Reallocation	Change (95% CI)	Change (95% CI)	Change (95% CI)	Change (95% CI)
**+** Sleep duration; **−** MVPA	-0.033 (-0.065; -0.001)	-0.026 (-0.046; -0.005)	-0.049 (-0.079; -0.019)	0.025 (0.006; 0.044)
**+** Screen time; − MVPA	0.026 (-0.005; 0.057)	0.024 (0.005; 0.044)	0.019 (-0.009; 0.048)	0.072 (0.054; 0.090)
**+** Sleep duration; − Screen time	-0.065 (-0.080; -0.050)	-0.054 (-0.065; -0.044)	-0.075 (-0.093; -0.057)	-0.052 (-0.064; -0.039)
**+** MVPA; − Screen time	-0.037 (-0.054; -0.020)	-0.030 (-0.040; -0.020)	-0.035 (-0.054; -0.016)	-0.054 (-0.066; -0.043)
**+** Screen time; − Sleep duration	0.062 (0.047; 0.076)	0.053 (0.043; 0.063)	0.072 (0.054; 0.089)	0.050 (0.038; 0.062)
**+** MVPA; − Sleep duration	0.031 (0.015; 0.048)	0.027 (0.017; 0.038)	0.044 (0.025; 0.063)	0.000 (-0.012; 0.012)

MVPA, moderate-to-vigorous physical activity; CI, confidence interval.

All models are adjusted for race/ethnicity, BMI z-scores, baseline movement behaviour composition, and baseline depressive symptoms.

*These changes are expressed as proportions of the pooled SD. Reallocation of 60 minutes to one activity (+) from another activity.

(-) and resulting change in depressive symptoms, which could be either significant (decrease (-) or increase (+) while the 95% confidence intervals do not include value 1) or not significant (when the 95% confidence intervals include value 1).

## Discussion

Using compositional data analysis on a large and prospective cohort of adolescents, our results showed that the relative contribution of MVPA to the overall movement behaviour composition decreased over time among all subgroups, whereas the relative contribution of sleep duration decreased, and that of screen time increased among younger boys, younger girls, and older girls. Before and after adjusting for covariates, increasing sleep duration relative to the remaining behaviours (i.e. screen time and MVPA) was associated with lower depressive symptoms among all subgroups. Conversely, increasing screen time relative to the remaining behaviours (i.e. MVPA and sleep duration) was associated with higher depressive symptoms among all subgroups. Increasing MVPA relative to the remaining behaviours (i.e. screen time and sleep duration) was associated with lower depressive symptoms in older girls only. Results further indicated that predicted changes in depressive symptoms were strongest and most beneficial when replacing 60 minutes of screen time by 60 minutes of sleep duration across all subgroups.

### Change in movement behaviour composition

Our results showed that there was a decrease in the relative contributions of MVPA and sleep duration to the overall movement behaviour composition and an increase in the relative contribution of screen time at 1-year follow-up among all subgroups, except older boys. In the latter subgroup, the relative contribution of MVPA significantly decreased, whereas the relative contributions of both screen time and sleep duration to the overall movement behaviour composition has significantly increased over time. The overall pattern is consistent with current literature indicating that as adolescents get older they become less active and well versed into modern sedentary activities [42,43]. In a large nationally representative sample of the US population, Yang et al. [42] found an increase in the prevalence of computer use during leisure time and the total sitting time among adolescents. They also found that the prevalence of sitting watching television or videos for 2 hours or more per day remained high and stable from 2001 through 2016 in this age group [42]. In parallel, recent research has shown that heavy screen time is associated with short sleep duration among adolescents [44,45]. It is possible that a simple time displacement of sleep duration by screen time, particularly around sleep time, at least in part explains this association. In addition, late night screen time use could shift the circadian rhythm towards a later midpoint of sleep and increase mental and physiological arousal before bedtime, which could delay sleep onset [46]. It is also possible that screen time results directly in short sleep duration via melatonin suppression by the blue light of screen devices, thus resulting in a desynchronization of the circadian rhythm [47]. However, among older boys, the relative contributions of MVPA and screen time follow the same pattern as in the other subgroups, except for a negligible 1% increase in the relative contribution of sleep duration. Regardless, there is an aging effect that should be acknowledged, according to which it could be normal for physical activity and sleep to decline as teenagers age. Future studies with a longer follow up are necessary to examine change in movement behaviours over time across different subgroups. Nevertheless, our findings underscore the need for interventions to encourage active living and sufficient sleep duration among adolescents.

### Association between change in movement behaviour composition and change in depressive symptoms

Our results showed that increasing sleep duration relative to remaining behaviours was associated with lower depressive symptoms whereas increasing screen time relative to the remaining behaviours was associated with higher depressive symptoms among all subgroups. Increasing MVPA relative to the remaining behaviours was associated with lower depressive symptoms in older girls only. The results of this study are consistent with recent studies indicating that sleep duration and recreational screen time are stronger predictors of mental health among adolescents than physical activity [48–51]. Using a representative sample of over 10,000 Canadian middle and high school students, we have previously found that meeting the screen time recommendation alone or the sleep duration recommendation alone were strongly associated with lower odds of suicidal ideation and suicide attempts, particularly among girls [49]. Similarly, Walsh et al. [50] and Guerrero et al. [48] found that meeting the screen time and sleep duration recommendations were strongly associated with better cognitive function and less impulsivity in a representative sample of US children, respectively, while meeting physical activity recommendations was not. However, these studies were limited by their cross-sectional design. Studies looking at the prospective association between changes in adherence to movement behaviours and mental health indicators have found that adherence to the sleep duration recommendation was the most consistent predictor of lower depression symptoms and flourishing among adolescents [13,52]. The present study extends previous evidence by using compositional data analysis, which provides estimates that are fully adjusted for all time use and permit an exploration of the combined associations of the different movement behaviours.

The finding that increasing MVPA relative to the remaining behaviours was associated with lower depressive symptoms in older girls only is interesting and deserves further investigation. The benefits of regular physical activity on adolescent mental health are well known [53]. Boys are known to be more active than girls, and the prevalence of adherence to MVPA decreases with age [54–56]. On the other hand, girls are well known to have more mental health problems than boys and experience of mental health problems increases with age [57]. Sampasa-Kanyinga et al. [49] have recently shown that older girls had the lowest prevalence of adherence to the physical activity recommendation, and reported more suicidal ideation and suicide attempts than older boys, young girls, and younger boys. In parallel, research has shown that physical activity is prospectively associated with lower depressive symptoms among adolescents [58]. Moreover, physical activity has been identified as an effective first-line treatment for mild-to-moderate depression, improving depressive symptoms to a comparable extent as pharmacotherapy and psychotherapy [59,60]. Our results suggest that increasing physical activity could be a good behavioural intervention to prevent depressive symptoms among older girls. It is difficult to speculate on what could explain such difference given the novel nature of our findings. It is possible that MVPA could be more beneficial to older girls given their greater drop in activity levels and vulnerability to depressive symptoms. Future research is needed to better understand why and how increasing MVPA relative to the remaining behaviours is associated with lower depressive symptoms among older girls only.

### Compositional isotemporal substitution

Our isotemporal substitution estimates suggest that decreasing screen time by 60 minutes/day and replacing that time with 60 minutes of additional sleep is associated with the largest changes in depressive symptoms across all subgroups. These findings are interesting because research has shown that heavy screen time occurs in tandem with short sleep duration among adolescents [44,45]. Our results suggest that getting sufficient sleep duration and decreasing recreational screen time could be good behavioural targets to prevent depressive symptoms among adolescents. However, this is challenging, because with rapid progress in information communication and technology, screen time, particularly time spent using electronic media and video games, have become omnipresent in the daily life of most adolescents [61,62]. In parallel, heavy use of electronic media, such as use of social networking sites, particularly around bedtime has been shown to result in short sleep duration [63]. It is possible that heavy screen time displaces sleep duration, as it could shift circadian timing to a latter point. It is also possible that the blue light from electronic media explains short sleep duration among adolescents [63]. As such, it is possible that effective interventions that reduce screen time also improve sleep duration among adolescents. This is particularly important because short sleep duration is increasingly widespread among adolescents [64]. This supports the need for the development, implementation and evaluation of sleep promotion interventions (especially within schools where students could be provided equitable access), as school-based sleep programs have previously shown potential long-term benefits [65].

### Strengths and limitations

This study has several strengths worth mentioning. First, it uses a large and linked sample of adolescents, thus supporting temporality between movement behaviours and depressive symptoms. Second, we used compositional data analysis, which has been identified as the most appropriate method to account for the co-dependent nature of movement behaviour data [15]. Third, our analyses are stratified by age groups and gender. Differences by subgroup help inform the development of tailored interventions intended to prevent depressive symptoms among adolescents (e.g., by finding ways to increase MVPA levels in older girls).

Our study also has several limitations that should be recognized. First, the analyses are based on self-reported measures, thus subject to desirability and recall biases. For example, a recent study found that self-reported and accelerometer-measured estimates of physical activity are poorly correlated in a representative sample of Canadian adolescents, and that youth self-report considerably more physical activity than what they accumulate on the accelerometer [66]. Second, our study did not include light-intensity physical activity, because it is not available in COMPASS, but it is an important component of daily movement behaviour. It is possible that light physical activity is favourably associated with mental health outcomes. For example, a prospective study using repeated device-based measures of physical activity found that light physical activity was more consistently associated with lower depressive symptoms in adolescence than total physical activity and MVPA [24]. Future studies including this component are needed to examine how the full 24-hour movement behaviours impact upon depressive symptoms. Third, it is important to note that we did not include non-screen sedentary behaviours (e.g., reading a book, driving a car) because there were not available. Finally, it is possible that the strength of the associations reported herein are biased because the survey excluded adolescents who dropped out of school, adolescents experiencing homelessness, or institutionalized adolescents. These excluded groups are well known to have greater risk of engaging in health compromising behaviours and experiencing mental health problems [67,68].

### Conclusion

To the best of our knowledge, the present study is the first to examine prospective associations between movement behaviours and depressive symptoms among adolescents within a compositional data analysis framework among different age/gender subgroups of adolescents. Results suggest that increased sleep duration and reduced screen time are important determinants of lower depressive symptoms among adolescents. Increased awareness among different stakeholders, including parents, schools, health services providers, and adolescents themselves about the potential value of adequate sleep and engaging in less recreational screen time may help prevent mental health problems in this age group. School-based interventions promoting healthy active living and sufficient sleep are also needed as behavioural strategies to prevent mental health problems among adolescents. Future research is also needed to better understand some of the differences among subgroups.
